# Evodiamine Attenuates Experimental Colitis Injury Via Activating Autophagy and Inhibiting NLRP3 Inflammasome Assembly

**DOI:** 10.3389/fphar.2020.573870

**Published:** 2020-11-09

**Authors:** Wenwen Ding, Zhiquan Ding, Yong Wang, Yan Zhu, Qi Gao, Wangsen Cao, Ronghui Du

**Affiliations:** ^1^ Jiangsu Key Laboratory of Molecular Medicine, Medical School of Nanjing University, Nanjing, China; ^2^ State Key Laboratory of Analytical Chemistry for Life Science, Nanjing University, Nanjing, China

**Keywords:** evodiamine, colitis, NLRP3 inflammasome, autophagy, ASC

## Abstract

Autophagy and NLRP3 inflammasome were associated with the process of colitis. Drugs targeting NLRP3 inflammasome and autophagy to treat colitis are absent, and they are urgently required. Herein, we examine the effect of evodiamine, extracted from the fruit of *Evodiae Fructus*, on experimental colitis induced by dextran sulfate sodium and exposit whether evodiamine effects on autophagy and NLRP3 inflammasome. Our data indicated that colitis was ameliorated by evodiamine, including the improvement of mice body weight, colon length, histopathologic score, and the disease activity index. We also observed that evodiamine restrained the formation of the NLRP3 inflammasome by inhibiting the apoptosis-associated speck-like protein oligomerization and caspase-1 activity in THP-1 macrophages. Our results demonstrated evodiamine inhibit NLRP3 inflammasome activation via the induction of autophagosome-mediated degradation of inflammasome and the inhibition of NFκB pathway, which synergistically contribute to the effect of evodiamine in colitis. It indicates the potential use of evodiamine in inflammatory bowel diseases treatment.

**FIGURE 7:**
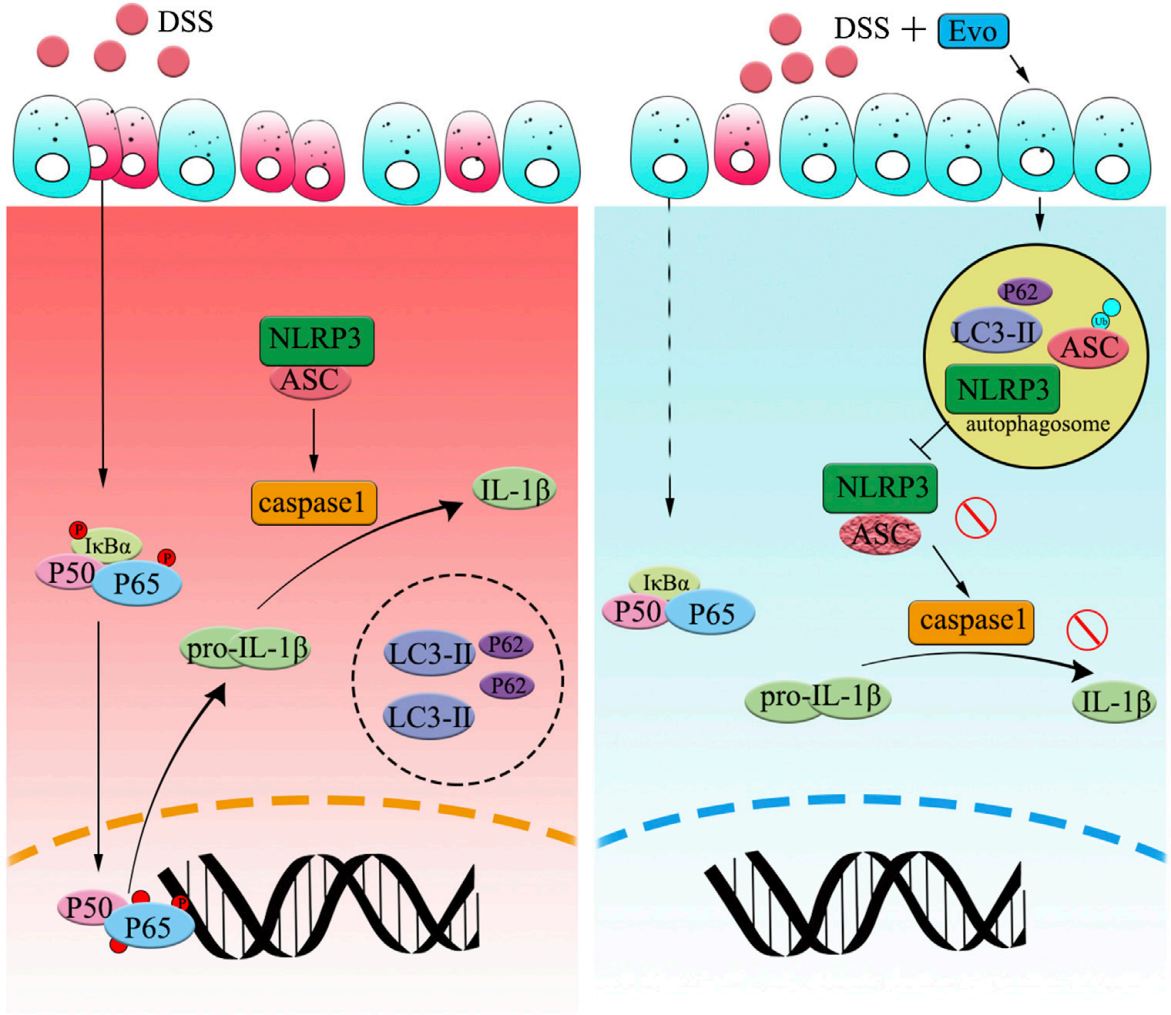
**Graphical Abstract** | Evodiamine ameliorates ulcer colitis through the activation of autophagy and the inhibition of NLRP3 inflammasome assembly. Under the condition of acute inflammation induced by DSS, NLRP3 inflammasome is activated, autophagy was blocked, thereby the clearance of pro-inflammatory is impaired. Evodiamine activates autophagy, leads to autophagosomal degradation of pro-inflammatory cytokine upstream of the NFκB signaling cascade. Moreover, the assembly of NLRP3 inflammasome was also suppressed by evodiamine due to the inhibition of ASC. As a result, the production of inflammatory cytokine such as IL-1β decreased and colitis was attenuated.

## Introduction

Ulcerative colitis (UC), a risk factor of colon cancer (Rogler, 2012), mainly occurs at the colon site and spreads from rectum to proximal colon and even ileum, is a chronic inflammatory disease with a high recurrence risk (Wei et al., 2017). The main character of UC is the massive infiltration of neutrophils, monocytes and lymphocytes in the intestinal tract and production of pro-inflammatory cytokine (Baumgart and Sandborn, 2007; Salim and Soderholm, 2011). Inflammatory cells infiltration is mostly induced by the destroyed epithelial barrier (Zaki et al., 2011). It is also widely accepted that inappropriate mucosal immune system response contributes to the production of pro-inflammatory cytokine (Salim and Soderholm, 2011). During the inflammatory process, the damage was recognized by inflammatory cells via a series of damage sensing receptors (DSRs) (Ting et al., 2008), several components of the NOD-like receptor (NLR) family are regarded as vital regulators of secretion of cytokines (Jin and Flavell, 2010) since danger and pathogen-associated signals were rapidly responded by the inflammasome multiprotein complexes.

In several inflammatory diseases, the excessive activation of inflammasomes such as NLRP3 inflammasome is found (Chen H. et al., 2017). NLRP3, is one of NLR families and interacts with the adaptor protein, apoptosis-associated speck-like protein (ASC). NLRP3 inflammasome is a multiprotein complex which consists of NLRP3, ASC, and pro-caspase 1. NLRP3 inflammasome mediated the production of IL-1β and IL-18 via a classical two-step process (Wang et al., 2016). In the first step, priming mediated by NF-κB activation, induces the synthesis and accumulation of the precursor proteins, including pro-IL-1β, pro-IL-18, and NLRP3. After NLRP3 expression prime with the stimulation of LPS, caspase-1 cleavage was activated by a secondary signal ATP. Once NLRP3 inflammasome is activated, IL-1β and IL-18 will be produced followed by the activation of the cleaved-caspase-1 (Jin and Flavell, 2010). Xia et al. has reported that NLRP3^−/−^ mice ameliorated the severity of dextran sulfate sodium (DSS)-induced colitis, compared with wild type mice (Xia et al., 2019). The pathogenic roles of NF-κB and NLRP3 in the progression of UC also imply that targeting both signaling pathways is a promising strategy for effective therapeutics for UC (Sun et al., 2015).

Potential damage and pathogenic stimuli in inflammatory responses were also fine controlled by autophagy (Deretic et al., 2013). Autophagy (macroautophagy) activation is a multistage process which are composed by the formation of a membrane phagophore, the elongation (double-membrane autophagosomal structures) and the maturation step (fusion with late endosomes and lysosomes) (Hamasaki and Yoshimori, 2010).

Autophagy and inflammasome are two different fundamental cellular responses. Autophagy maintains cellular homeostasis by degrading cytoplasmic contents, including compromised organelles and aggregated proteins, and damaged organelles (Ma et al., 2013; Ryter et al., 2013) in a potent lysosomal degradation pathway. Responding to dangerous stimuli, inflammasome activates caspase-1 to secrete inflammatory cytokine. Autophagy and inflammasome pathways are closely linked and mutually regulated (Nakahira et al., 2011; Shi et al., 2012). In macrophages, inflammasome and autophagy can be activated simultaneously by a variety of stimuli. Deretic et al. has found that specific inflammasome sensors can induce autophagy and meanwhile, inflammasome activation was negatively regulated by autophagy (Dupont et al., 2011; Shi et al., 2012). Autophagosomes can degrade inflammasome in the presence of p62, the selective autophagic receptor. To consider the mutual relationship between autophagy and inflammasomes is critical in the treatment of UC.

Corticosteroids, 5-aminosalicylic acid, immunomodulators, and other anti-inflammatory and immunosuppressive biological agents, are commonly used to alleviate UC in patients. Clinical studies demonstrated that repeated use of these agents led to serious side effects, including osteoporosis, hypertension, diabetes, and infections, etc. (Tursi et al., 2010). It is urgent to find a novel drug with few side effects for UC patients.

Evodiamine, a quinazoline alkaloidal, is extracted from the fruit of *Evodiae Fructus* (*Evodia rutaecarpa* Benth., Rutaceae) which is also named “Wu-Zhu-Yu.” Evodiamine is widely used in Traditional Chinese Medicine because it exhibits various effects such as antitumor growth, anti-anoxic, anti-metastatic, antinociceptive, and anti-inflammatory (Liu A. J. et al., 2013; Wang et al., 2015). Studies have demonstrated that evodiamine has a potential therapy for diarrhea, abdominal pain, and ulcer (Zhao et al., 2015). Zhang’s lab reported that evodiamine prevented dextran sulfate sodium-induced murine experimental colitis via the regulation of NF-κB and NLRP3 inflammasome (Shen et al., 2019). However, the link between inflammasome and autophagosome remains unclear. In this study, we addressed to reveal the effect of evodiamine on inflammasome and autophagy.

## Materials and Methods

### Chemicals and Reagents

Evodiamine (>98% HPLC) was purchased from Chengdu Puruifa Biotechnology Co., Ltd. (Chengdu, China) (Shen et al., 2019). DSS (36–50 kDa) was obtained from MP Biomedicals (Aurora, OH). Phorbol 12-myristate 13-acetate (PMA), lipopolysaccharide (LPS), adenosine triphosphate (ATP), DSS (5 kDa), 5-aminosalicylic acid (5-ASA) and 4′,6-diamidino-2-phenylindole (DAPI) were bought from Sigma–Aldrich (St. Louis, MO, United States). ELISA kits for murine MPO, IL-1β and IL-18 were purchased from Multi Sciences Biotech Co., Ltd. (Hangzhou, China), ELISA kits for human IL-1β and IL-18 were obtained from Excell Biotech Co., Ltd. (Taicang, China). RPMI-1640 medium, fetal bovine serum (FBS) (Gibco, United States), anti-NLRP3, anti-Caspase1 and anti-IL-1β antibodies were purchased from ABclonal Technology (ABclonal, Boston, United States), anti-ASC, anti-cleaved caspase1 and anti-LC3 antibodies were bought from cell signaling technology (CST, Danvers, United States), anti-ASC (mouse) antibody was purchased from Santa Cruz Biotechnology (Santa Cruz, CA, United States), anti-CD11b and anti-LAMP-1 antibodies were obtained from Abcam technology (Cambridge, United Kingdom), anti-GAPDH and anti-P62 antibodies were purchased from Proteintech group (Chicago, United States), HRP-conjugated secondary antibody, Alexa Fluor-488 conjugated goat anti-rabbit or anti-mouse IgG (H + L) secondary antibodies and Alexa Flour-594 conjugated goat anti-rabbit IgG (H + L) secondary antibody were bought from Invitrogen (Carlsbad, United States).

### Cell Culture

Human THP-1 cells were grown in RPMI-1640 medium, supplemented with 10% (v/v) fetal bovine serum, 100 mg/ml streptomycin and 100 U/ml penicillin under a humidified environment with 5% CO_2_ at 37°C.

### Animal Experiments

C57BL/6J mice (male, 6–8 weeks old, 18–22 g) were purchased from Model Animal Research Center of Nanjing University (Nanjing, China). All mice were housed in the experimental animal facility, water and food were available *ad libitum*. Mice were randomly assigned to control (vehicle; 0.5% sodium carboxymethylcellulose, CMC-Na), DSS-treated, evodiamine-treated (20, 40, and 60 mg/kg), evodiamine-only (60 mg/kg) and 5-ASA-treated (100 mg/kg, positive control) groups. Acute colitis was induced by 3% DSS (36–50 kDa) in drinking water for 7 days, then change treated water to normal water for another continuously 3 days before sacrifice (Okumura et al., 2016; Chen H. et al., 2017). Evodiamine and 5-ASA dissolved in the sodium solution of carboxymethyl cellulose (CMC-Na, 0.5%) and treated by oral gavage once a day from day 1 to day 10. All animal welfare and experiment procedures were carried out rigid according to guideline of European Directive 2010/63/EU and approved by the Nanjing University Animal Care and Use Committee (NJU-ACUC).

### Disease Activity Index and Histological Analysis

The severity of colitis was evaluated by colon length, the DAI, histologic analyses, and MPO activity (Tasaka et al., 2015). Body weight, fecal blood loss and stool consistency were monitored daily. Briefly, the disease activity index (DAI) was calculated as the sum of three parameters as follows: body weight loss (0, ≤1%; 1, 1–5%; 2, 5–10%; 3, 10–15%; 4 ≥ 15%), diarrhea (0, normal; 2, loose stools; 4, watery diarrhea) and blood in the stool (0, no bleeding; 2, slight bleeding; 4, gross bleeding) (Song and Park, 2014), also shown in Table 1. After animals were sacrificed, part of the colon was dissected. To evaluate the degree of morphometric inflammation, the length between the ileocecal junction and the anal verge were measured. The distal part of colon was fixed in 4% paraformaldehyde, embedded in paraffin and sliced into 5 μm-thick sections. The sections were routinely stained with hematoxylin and eosin (H&E) (Hayashi et al., 2014). Formalin-fixed hematoxylin tissue sections were evaluated by an experienced pathologist microscopically in a blinded way. Severity of inflammation, crypt damage and ulceration were simultaneously evaluated (Wu et al., 2012).

**TABLE 1:** Disease Activity Index assessment criteria for dextran sodium sulfate-induced colitis model.

Assessment criteria	Score	Description
Body weight loss	0	≤1%
	1	1–5%
	2	5–10%
	3	10–15%
	4	≥15%
Stool consistency	0	Normal
	2	Loose stools
	4	Diarrhea
Grossing bleeding	0	Negative
	2	Positive
	4	Gross positive

### Myeloperoxidase Analysis

Myeloperoxidase (MPO) level was determined by ELISA to evaluate neutrophil infiltration into inflamed colonic mucosa. Briefly, colon tissues were weighed and homogenized in cold phosphate buffer saline (PBS) for 5% colonic tissue homogenate (Mei et al., 2019). Supernatants were collected to determine MPO levels according to the manufacturer’s instructions after they were centrifuged at 12,000 *g* at 4°C for 15 min. The results were presented as absorbance per mg of tissue.

### Cytokine Analysis by Enzyme-Linked Immunoassay

Colonic tissues of the mice were weighed and homogenized in cold PBS for 5% colonic tissue homogenate. To obtain the supernatants, homogenate was centrifuged at 12,000 *g* at 4°C for 15 min. Protein concentration in the supernatants was determined by BCA protein assay, and cytokines in the colonic tissue homogenate were analyzed by ELISA kits. For *in vitro* assay, THP-1 cells were seeded in 12-well plates and the supernatants were harvested after stimulation. IL-1β and IL-18 were detected using ELISA kits according to the manufacturer’s instructions.

### Immunofluorescence Histochemistry

Paraffin-embedded colonic tissues were stained with CD11b antibody to analyze inflammatory cell infiltration. The sections were deparaffinized in xylene and rehydrated in a decreased alcohol gradient and washed with ddH_2_O. After unmasking antigens, colon sections were blocked with 5% BSA and incubated with anti-CD11b antibody (1:200) at 4°C overnight, and FITC labeled secondary antibody at room temperature for 1 h. Finally, sections were counterstained with DAPI. Images were acquired by an Olympus FV3000 confocal microscope.

### Western Blot Analysis

The distal colon was removed, washed with ice-cold PBS, and homogenized in lysis buffer containing 1% Triton X-100, 10 mM Tris (pH 7.4), 1 mM EDTA, 1 mM EGTA, 150 mM NaCl, protein inhibitor cocktail (1:100) and Phenylmethylsulfonyl fluoride (PMSF) by ultrasonic splitting on ice. After centrifugation at 12,000 *g* for 30 min at 4°C, the supernatants were collected and protein concentrations were determined using a BCA protein assay kit (Pierce, Rockford, IL), then were stored at −80°C till for use. The lysates were mixed with Laemmli sample buffer and heated at 95°C for 5 min. The samples were subjected to electrophoresis on 10% SDS-polyacrylamide gels and transferred electrophoretically to PVDF membranes (Millipore, Billerica, MA). The membranes were blocked with TBST (0.1% Tween 20 in Tris-buffered saline) containing 5% skimmed milk at room temperature for 2 h, incubated with the primary antibodies at a predetermined dilution at 4°C overnight. Subsequently, the membranes were washed three times with TBST, incubated with the horseradish peroxidase (HRP)-conjugated secondary antibody at room temperature for 2 h, and then the membranes were visualized with ECL detection reagent.

### ASC Oligomerization Assay

THP-1 cells were seeded in 6-well plates and stimulated as indicated. Cells were harvested by scraping in 1 ml cold PBS containing 2 mM EDTA, and centrifuged at 1,500 *g* for 5 min at 4°C. Pellets were re-suspended the in 500 μl cold buffer A (20 mM HEPES-KOH, pH 7.5, 10 mM KCL, 1.5°mM MgCl_2,_ 1 mM EDTA, 1 mM EGTA, and 320 mM sucrose) and cell were lyzed by shearing 30 times through 21-gauge needle. Subsequently, the lysates were centrifuged at 1,800 *g* at 4°C for 8 min to remove the bulk nuclei and unbroken cells (Okada et al., 2014). The supernatants were diluted in CHAPS buffer [20 mM HEPES-KOH (pH 7.5), 5 mM MgCl_2_, 0.5 mM EGTA, 0.1 mM PMSF, and 0.1% CHAPS] and centrifuged at 5,000 *g* for 8 min at 4°C to pellet ASC oligomers (Lorden et al., 2017). Then the pellets were resuspended in 50 μl CHAPS buffer with 2 mM disuccinimidyl suberate to cross-link at RT for 30 min and pelleted by centrifugation at 5,000 *g* for 8 min. The crosslinked pellets were collected for ASC oligomerization assay by immunoblotting (Chen L. et al., 2017).

### Immunofluorescence Cytochemistry

The differentiated THP-1 cells were cultured on coverslips in 24-well plate and treated with LPS (100 ng/ml, 3 h), ATP (1 mM, 1 h) (Qin et al., 2016) and evodiamine (10 μM, 1 h). The cells were fixed in 4% paraformaldehyde for 30 min, washed for three times with PBS for 5 min, permeabilized with 0.2% Triton X-100 for 10 min, washed for three times, blocked with 5% BSA for 2 h (Wang et al., 2015). Cells were incubated with the primary anti-LC3 (1:150), anti-LAMP-1 (abcam, 1:100), anti-caspase1 (Proteintech, 1:150) or/and anti-ASC (CST, 1:200) overnight at 4°C. Washed for three times with PBST, cells were incubated with Alexa Flour 488 fluorescein-conjugated secondary antibody or the Alexa Flour 594 fluorescein-conjugated secondary antibody for 2 h (Thomas et al., 2010). The coverslips were counterstained with DAPI and images were taken by an Olympus FV3000 confocal microscope.

### Statistical Analysis

Statistical significance for multiple comparisons was performed by one-way ANOVA and Newman-Keuls analysis using Prism 5.0 (GraphPad Software). A *p* value <0.05 was considered statistically significant. All data are indicated as means ± SEM and from at least three independent experiments.

## Results

### Experimental Ulcerative Colitis Injury Was Attenuated by Evodiamine

**FIGURE 1:**
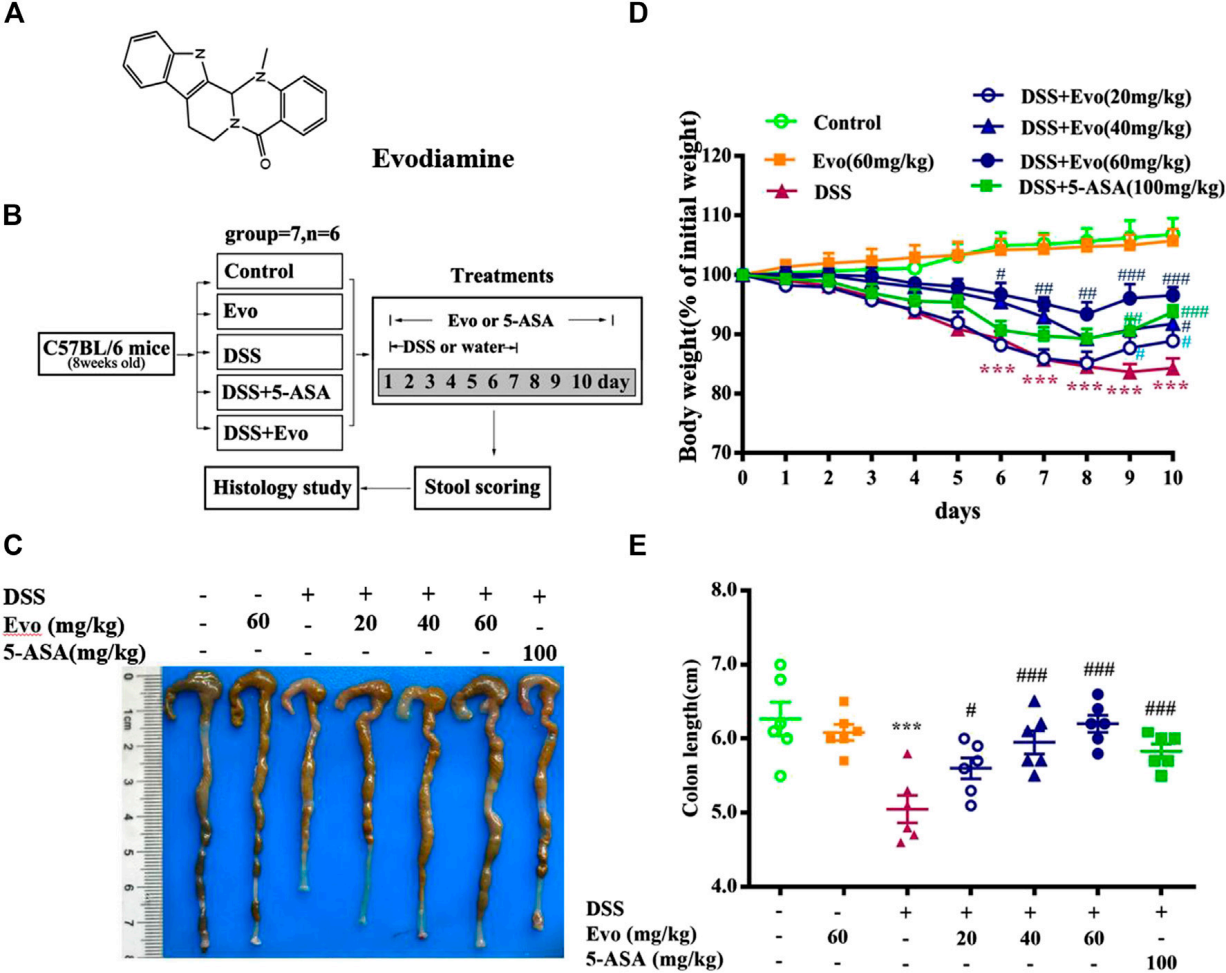
Evodiamine attenuated DSS-induced experimental ulcerative colitis injury in mice. Ulcerative colitis was induced by 3% DSS for 7 days and replaced by water for subsequent 3 days. Evodiamine and 5-ASA were administered once daily for 10 days by oral gavage, respectively. **(A)** Chemical structure of evodiamine. **(B)** A scheme of experimental design *in vivo*. **(C)** Macroscopic appearance of colon. **(D)** Body weight. **(E)** Colons length. Data are presented as mean ± SE (*n* = 6 for each group). *p < 0.05, **p < 0.01, and ***p < 0.001 compared with the control, #*p* < 0.05, ##*p* < 0.01, and ###*p* < 0.001 compared with the DSS group.

The structure of evodiamine is presented in Figure 1A. Colitis is characterized by significant diarrhea, loose feces, and visible fecal blood, resulting in a remarkable decrement in body weight (Liu W. et al., 2013). To evaluate the preventive effect of evodiamine on ulcer colitis, we performed the experimental colitis by DSS as showed in Figure 1B. Body weight of colitis mice induced by DSS decreased. After mice were treated by evodiamine for 10 days, colons length recovered, and the loss of body weight was improved (show in Figures.1C–E). To evaluate whether there is a toxic effect of evodiamine at the maximum concentration of 60 mg/kg, we administrated evodiamine alone to mice without DSS stimulation. There is no visible difference in body weight changes and colon length between evodiamine (60 mg/kg) and control mice. It suggested that the maximum dose of evodiamine was safe enough for treatment in this study.

**FIGURE 2:**
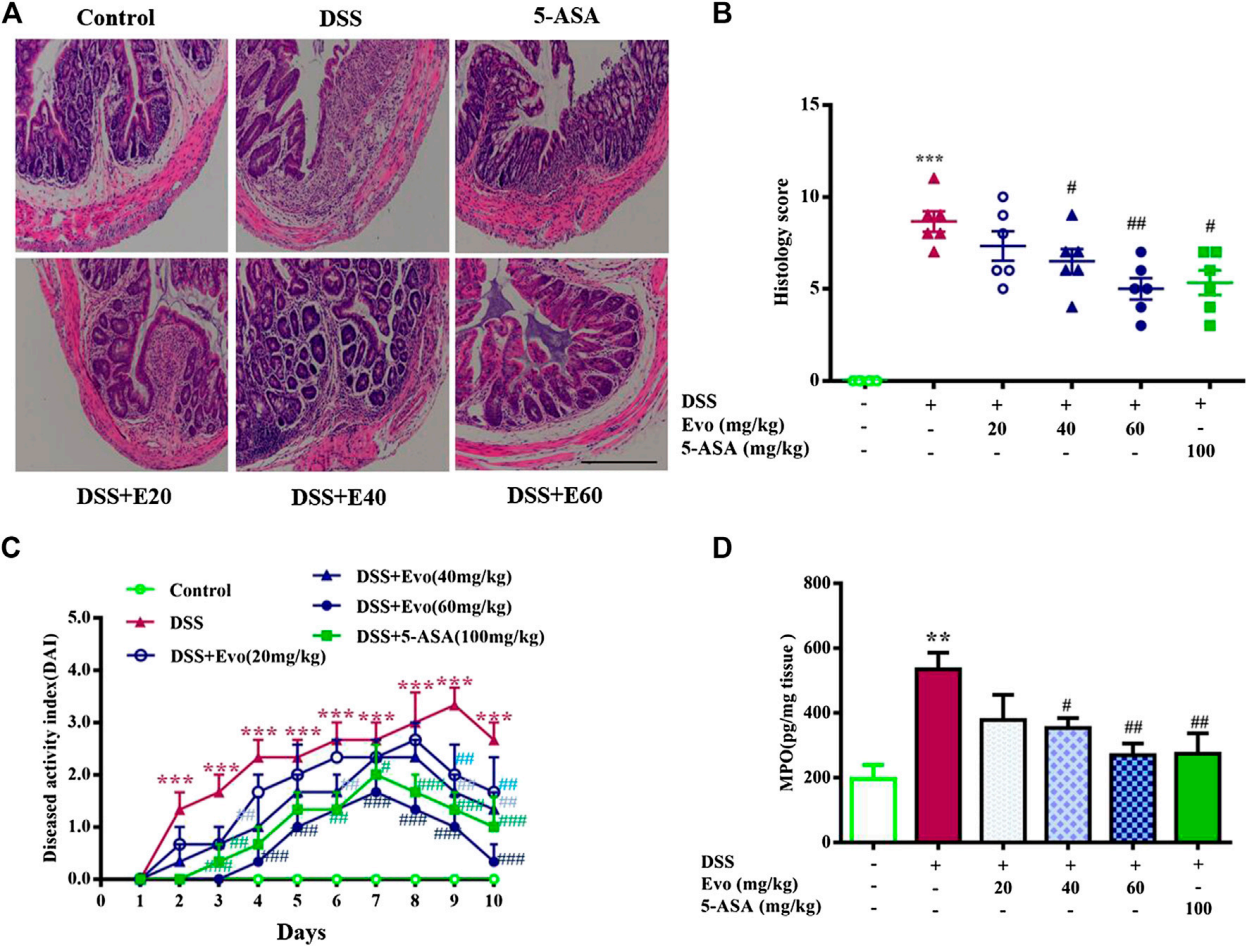
Evodiamine ameliorated colonic pathological damage in experimental ulcer colitis mice. **(A)** Paraffin-embedded colon sections were stained with H&E for assessment of epithelial damage. Original magnification 100×. **(B)** Histology score. **(C)** Disease activity index (DAI) scores were evaluated daily. **(D)** MPO level in colonic tissues. Data are presented as mean ± SE (*n* = 6 for each group). *p < 0.05, **p < 0.01, and ***p < 0.001 compared with the control, #*p* < 0.05, ##*p* < 0.01, and ###*p* < 0.001 compared with the DSS group.

To further confirm the anti-inflammatory effect of evodiamine on colitis, we observed pathological changes including mucosal damage, necrosis, the loss of tissue architecture, edema, and infiltration of inflammatory cells such as neutrophils and monocytes in colon tissues of mice by H&E staining and histological analysis. As shown in Figure 2A, pathological changes significantly improved by evodiamine dose-dependently (Figures 2A,B). DAI also deceased by evodiamine (Figure 2C). We continued to examine MPO levels, the important inflammatory index in colons of mice. MPO level in mice colon treated by evodiamine was much lower than that in the DSS group with the treatment of vehicle (Figure 2D). It suggested that attenuated ulcerative colitis injury by evodiamine resulted from the anti-inflammatory effect.

### Over-Activation of NLRP3 Inflammasome in Ulcerative Colitis Was Inhibited by Evodiamine

**FIGURE 3:**
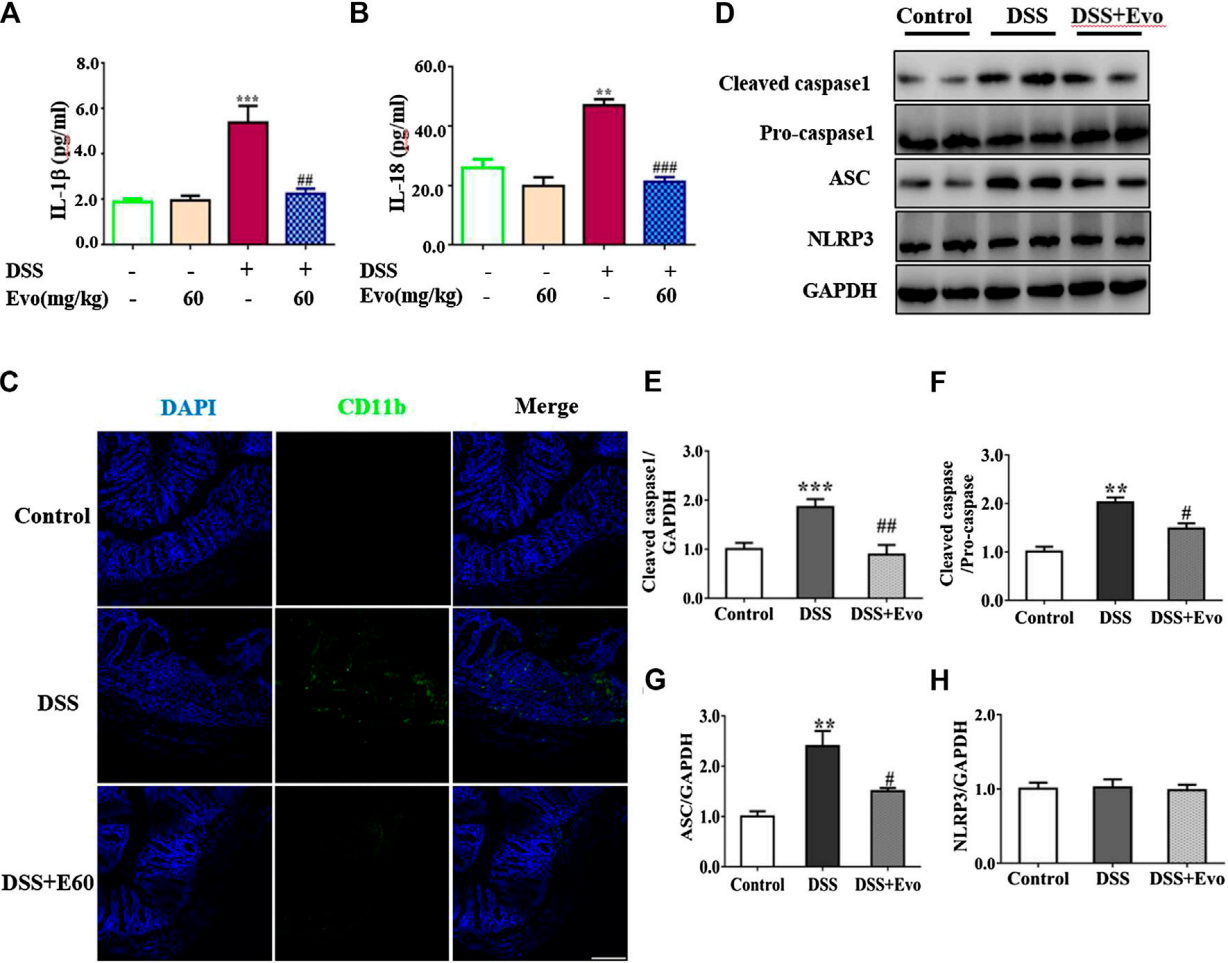
Evodiamine suppressed inflammation and decreased activity of NLRP3 inflammasome in DSS-induced mice. **(A,B)** IL-1β and IL-18 in colonic tissues were determined by ELISA, respectively. **(C)** Sections of colon were immunostained with DAPI (blue) and anti CD11b-FITC (green). Scale bars, 100 μm. **(D)** Expressions of cleaved caspase1, pro-caspase1, ASC and NLRP3 in colon were determined by immunoblot. **(E–H)** Quantification of **(D)** data were shown as mean ± SEM. **p < 0.01 and ***p < 0.001 compared with control, #*p* < 0.05, ##*p* < 0.05, and ###*p* < 0.001 compared with DSS-induced group.

It was reported that the occurrence of colitis is associated with the secretion of IL-1β and IL-18 (Zaki et al., 2010), our data also demonstrated that secretion of IL-1β and IL-18 increased in colitis mice colon. Levels of IL-1β and IL-18 in the serum with DSS stimulation were decreased by evodiamine (Figures 3A,B). To further confirm the anti-inflammatory effect of evodiamine, we examined macrophage infiltration in the colon sections using immunofluorescence. Macrophages indicated by CD11b were detected in the mucosa of colitis mice treated by vehicle, while little CD11b were observed in evodiamine-treated colonic mice samples. It demonstrated that macrophages infiltration was inhibited by evodiamine (Figure 3C).

Inflammatory cytokine IL-1β and IL-18 were active after their inactive cytoplasmic precursor pro-IL-1β and pro-IL-18 were cleaved by caspase-1 (Liu W. et al., 2013). We continued to examine whether cleaved caspase-1 can be regulated by evodiamine. Cleaved-caspase-1 in colitis mice colon was markedly decreased by evodiamine, so was the ratio of cleaved caspase one to pro-caspase 1 (shown in Figures 3D–F), it suggested that caspase-1 activation *in vivo* was inhibited by evodiamine. It was consistent with the decrement of IL-1β and IL-18.

After Pro-caspase-1 bound to ASC, it was autoclaved and the mature active form cleaved caspase-1 produced (Liu W. et al., 2013). Our data indicated that ASC in the colon was decreased after the intervention of evodiamine, while there was no significant alteration in NLRP3 expression (shown in Figures 3D,G,H).

### Decreased NLRP3 Inflammasome Activity Was due to the Inhibition of ASC Oligomerization and Inflammasome Assembly

**FIGURE 4:**
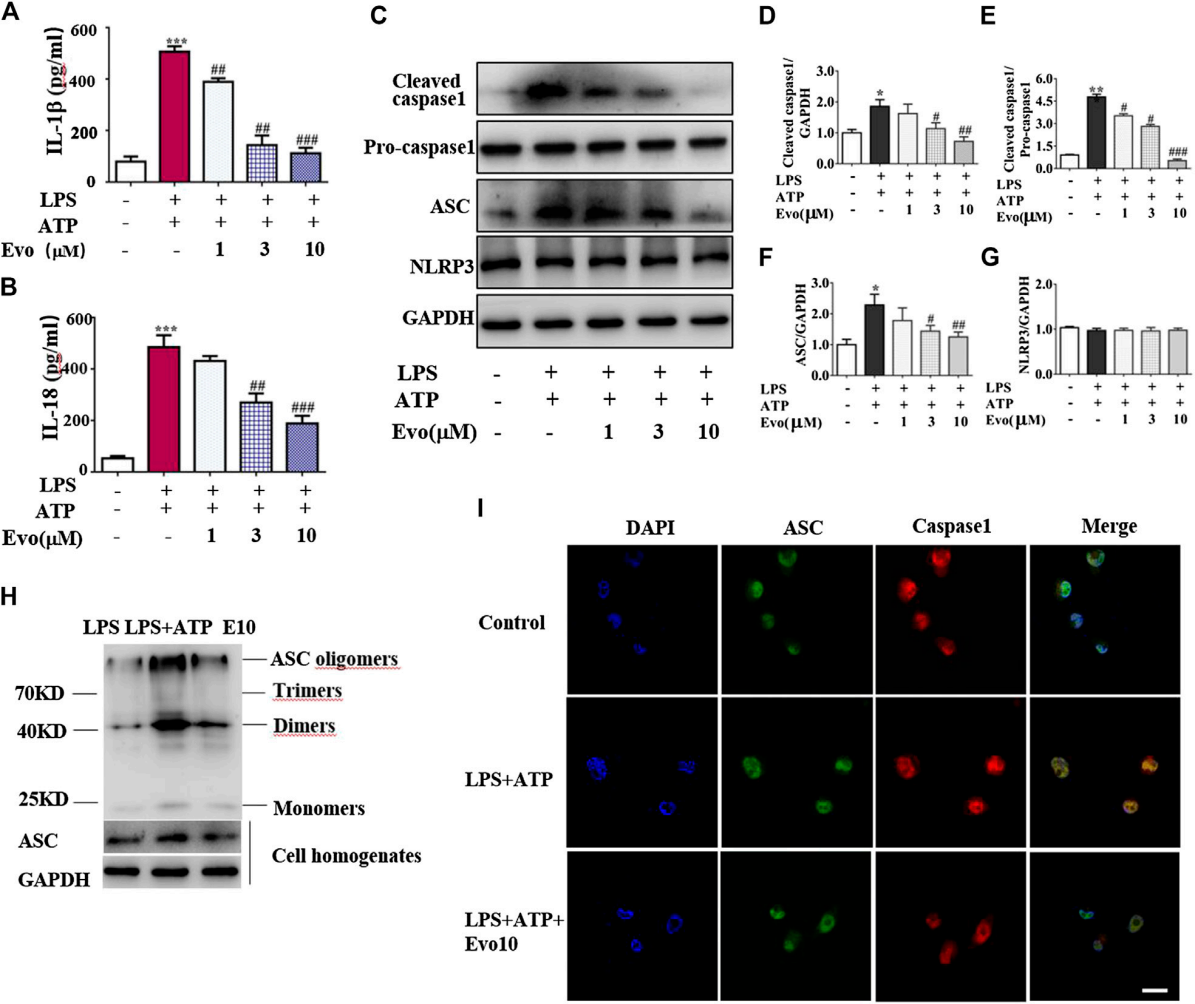
Evodiamine attenuated NLRP3 inflammasome activity in THP1 macrophages via inhibition of ASC oligomerization. Cells were stimulated with LPS (100 ng/ml) for 3 h and evodiamine for 1 h, then incubated with 5 mM ATP for 1 h. **(A,B)** Levels of IL-1β and IL-18 in THP1 cells were determined by ELISA. **(C)** Expressions of cleaved caspase1, pro-caspase1, ASC and NLRP3 in THP1 cells were determined by immunoblot. **(D–G)** Quantification of **Figure 3C**. **(H)** ASC oligomerization in THP1 cells were analyzed by immunoblot. **(I)** Immunofluorescence microscopy of THP1 stained with anti-ASC (green) and anti-caspase1 (red) antibodies. DAPI staining identified nuclei. Scale bar, 20 μm. The results are representative of at least three independent experiments and expressed as mean ± SD. *p < 0.05, **p < 0.05, and ***p < 0.001 compared with the control, #*p* < 0.05, ##*p* < 0.01, and ###*p* < 0.001 compared with the LPS + ATP group.

NLRP3 inflammasome was combined by ASC, NLRP3 and caspase-1 when cells face environmental stress. Under the stimulation of LPS and ATP, inflammasome was assembled, then caspase-1 was activated. As a result, the secretion of IL-1β and IL-18 was increased. To confirm whether evodiamine effects on NLRP3 inflammasome, we examined levels of IL-1β and IL-18 in THP1 cells by Elisa. As shown in Figures 4A,B, IL-1β and IL-18 remarkably increased in THP1 cells with the stimulation of LPS and ATP; the increment was significantly inhibited by evodiamine dose-dependently. To address how evodiamine act on NLRP3 inflammasome, we firstly examined NLRP3 expression using immunoblot. There is no remarkable alteration in NLRP3 in THP1 cells (Figures 4C,G), the level of ASC was up-regulated in the stimulation of DSS (Figures 4C,F). The decrement of IL-1β and IL-18 suggested the suppression of NLRP3 inflammasome, we further determine ASC oligomerization, the vital step for NLRP3 inflammasome activation (Lu et al., 2014; Chen L. et al., 2017).

As shown in Figure 4H, ASC oligomer was enhanced by ATP and LPS, and the increment was decreased by evodiamine. Bryan et al. reported that in response to ATP, ASC translocate from the nucleus, co-localizes with cytoplasmic caspase-1 (Bryan et al., 2009; Liu W. et al., 2013), then caspase-1 auto-cleaves and releases from the complex. Our immunoblot and immunofluorescence analysis showed that autocleavage of caspase-1 was inhibited by evodiamine (Figures 4C–E,I) so that colocalization of ASC and caspase-1 were interrupted, release of activated caspase-1 lessened. These data suggest that the assembly of NLRP3 inflammasome was inhibited by evodiamine via the suppression of ASC oligomerization.

### Autophagy Plays a Critical Role in the Inhibition of NLRP3 Inflammasome Induced by Evodiamine

**FIGURE 5:**
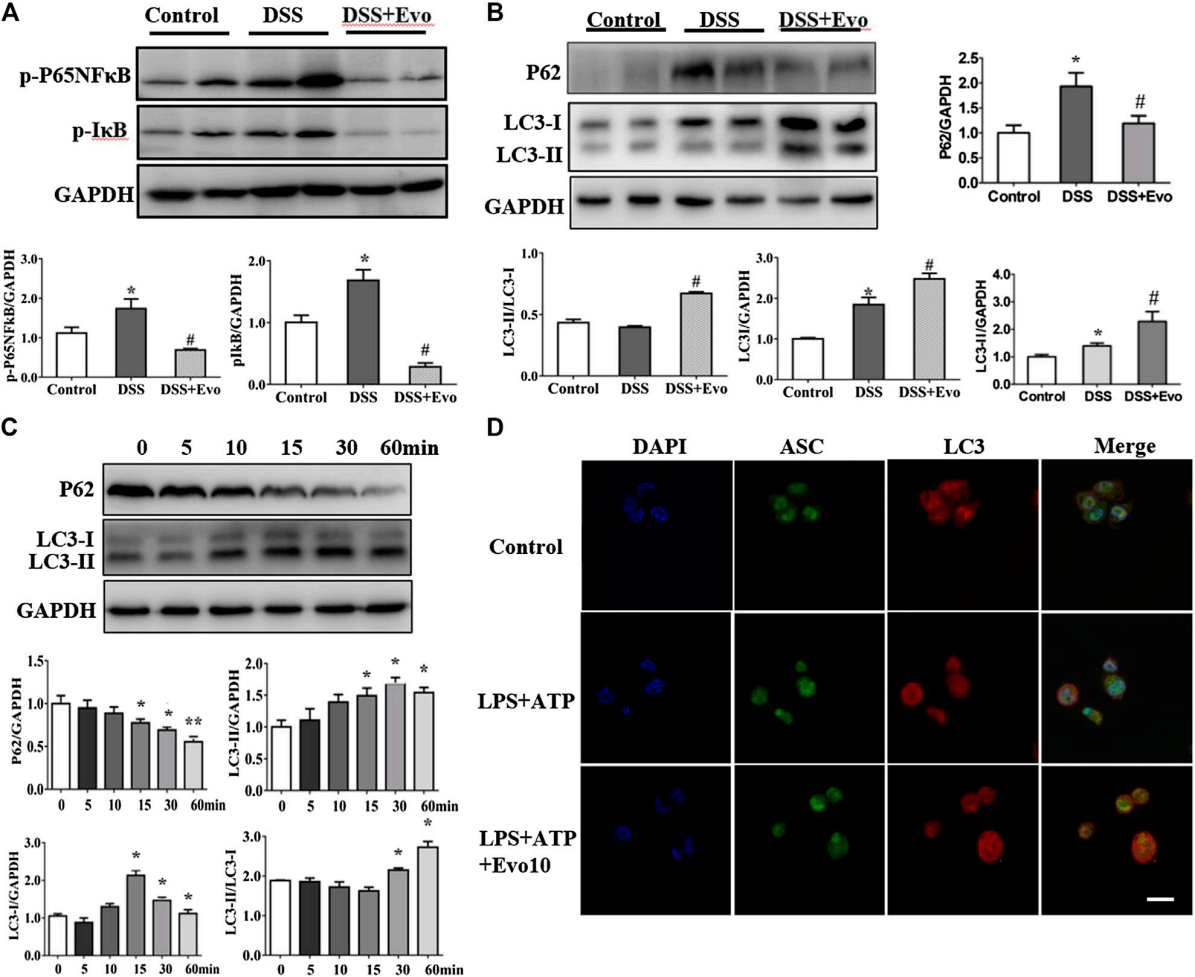
Evodiamine increased autophagic flux in colitis mice and differentiated THP1 cells. **(A)** Immunoblot analysis of p-P65, *p*-IκB expression in mice colon and quantification. **(B)** Immunoblot analysis of p62, LC3 expression in mice colon and quantification. **(C)** Expressions of p62 and LC3 in THP-1 cells stimulated with evodiamine (10 μM) at various time determined by immunoblot and quantification. **(D)** Representative confocal fluorescence microscopy images of THP-1 cells incubated with LPS and ATP in the presence or absence of evodiamine (10 μM) and immunostained for ASC (green) and LC3 (red). Nuclei were stained with the DAPI (blue). Scale bar, 10 μm. The data are representative of three independent experiments.

Autophagy and inflammasome interacts (Yuk and Jo, 2013; Kim et al., 2016). Pro-inflammatory cytokine upstream of the NFκB signaling cascade is in a state of inaction or reduced activity when autophagy activated. We examined the activity of p-p65NFκB and *p*-IκB which are critical protein in NFκB signaling cascade. As shown in Figure 5A, evodiamine decreased the phosphorylation of p65NFκB and IκB, it suggested that autophagy is likely activated. To further explore whether autophagy was able to be regulated by evodiamine, we investigated the degradation of p62 to confirm whether a complete autophagic flux occurred in evodiamine treatment condition. P62 protein in colon of colitis mice significantly decreased by evodiamine (shown in Figure 5B). Next, we examined the effect of evodiamine on p62 and LC3 at continuous time. Expression of p62 was suppressed by evodiamine time-dependently (shown in Figure 5C). At the same time, there is a slight increment in LC3-II expression induced by evodiamine. It suggested that autophagy in THP1 cells enhanced by evodiamine.

We also assessed LC3 expression in the cell lysates of mice colon using immunoblot analysis. LC3-I is cytosolic, while the hallmarks of autophagy, LC3-II is membrane- bound. We found that LC3-II protein levels increased in colitis mice colon after evodiamine treatment (Figure 5B). It implies that evodiamine increased autophagosome formation. We continue to address whether NLRP3 inflammasome and autophagy were simultaneously regulated by evodiamine. Co-localization of LC3 and ASC, which was able to be regarded as the intracellular sensor of NLRP3 was detected. As shown in Figure 5D, the overlap between ASC and LC3 were enhanced after the treatment of evodiamine. It suggested that the regulation of NLRP3 inflammasome induced by evodiamine might be via the intervention of autophagy.

### Evodiamine Inhibited NLRP3 Inflammasome via the Activation of Autophagy

**FIGURE 6:**
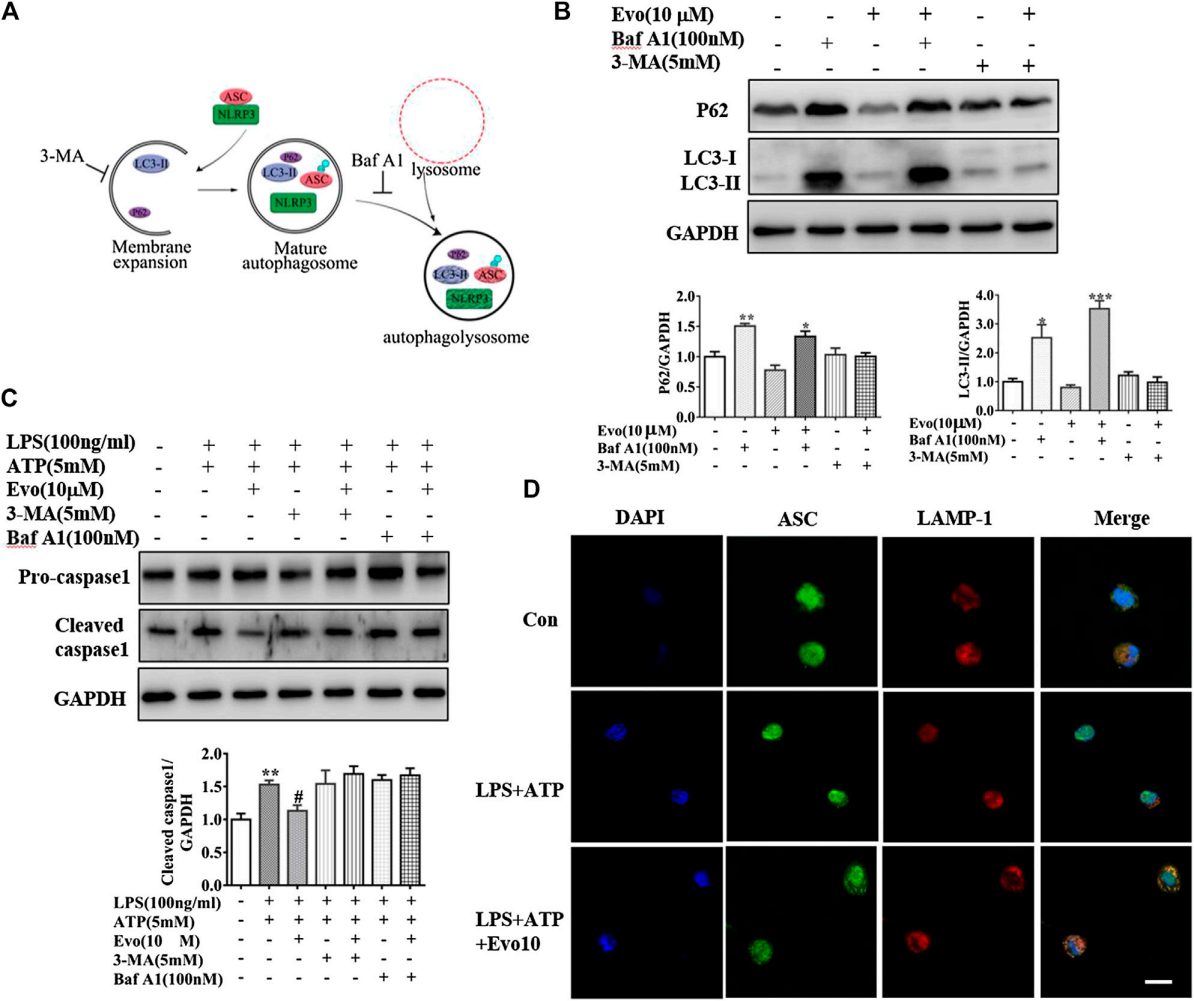
Evodiamine inhibited NLRP3 inflammasome via the activation of autophagy. **(A)** Sketch of autophagy flux. **(B)** Immunoblot analysis of p62 and LC3 in THP-1 cells which were incubated with evodiamine (10 μM) in the presence or absence of bafilomycin A (100 nM) or 3-MA (5 mM) and quantification. **(C)** Cleaved caspase one and pro-caspase one in THP-1 cells which were treated with bafilomycin A (100 nM), 3-MA (5 mM) alone or with Evo (10 μM) after stimulation with LPS (100 ng/ml) and ATP (5 mM) were determined by Immunoblot. **(D)** Confocal microscopy images of differentiated THP1 cells incubated with LPS and ATP with and without evodiamine, immunostained for ASC (green) and LAMP1 (red). Scale bars, 10 μm. The results are representative of at least three independent experiments and expressed as mean ± SD. *p < 0.05, **p < 0.01, and ***p < 0.001 compared with the control, #*p* < 0.05 compared with the LPS + ATP group.

Increment in autophagosome formation or decrement in autophagosome degradation cause enhanced autophagosome accumulation. To study how evodiamine regulate autophagy, we observed levels of LC3-II and p62 in THP1 cells co-incubated with bafilomycin A1 (Baf A1), blocking the fusion of autophagolysosome to inhibit the degradation, and 3-methyladenine (3-MA), inhibiting the autophagosome formation (Figure 6A). The expression of LC3-II increased when THP1 cells were incubated with evodiamine in the presence of Baf A1 (shown in Figure 6B). There is no significant effect on the expression of LC3-II induced by evodiamine in the presence of 3-MA. These data suggested that autophagosome degradation was significantly inhibited and the formation of autophagosome is slightly influenced by evodiamine.

We continued to investigate the effect of evodiamine on inflammasome activity after autophagy was regulated by Baf A1 and 3-MA. The expression of cleaved caspase1, the marker of inflammasome activation, was increased by LPS and ATP, the overexpression was suppressed by evodiamine. Cleaved caspase-1 could not be inhibited by evodiamine in the presence of autophagy inhibitors such as Baf A1 and 3-MA (Figure 6C). These data indicated that inflammasome activation was inhibited by evodiamine in an autophagy-dependent manner. Once autophagosome mature, the lysosomal and autophagosome fuse to form autophagolysosome, result in the degradation of autophagosome (Ganley et al., 2011). To confirm the autophagolysosome formation, we further detected the co-localization of ASC and LAMP-1. Shown in Figure 6D, in cells incubated with evodiamine, immunostaining indicated that ASC surrounded by LAMP-1 which is the lysosome marker, suggesting that autophagosomes fused with inflammasome components and lysosomal. These data demonstrated that the formation of autophagolysosome was enhanced and degradation was inhibited by evodiamine.

## Discussion

The ulcer colitis (UC) rises quickly these years, novel high-efficiency therapeutic agents for UC with few side effects are expected (Galli et al., 2011). In this study, we confirmed that evodiamine significantly attenuated experimental UC injury induced by DSS through the inhibition of NLRP3 inflammasome activation and inflammatory cytokine production. We showed that evodiamine inhibited the assembly of NLRP3 inflammasome for the first time. Furthermore, we found that evodiamine inhibited the degradation of autophagosome and the assembly of NLRP3 inflammasome is possibly dependent on autophagy. The link between autophagy and NLRP3 inflammasome intervened by evodiamine was firstly reported.

To confirm the effect of evodiamine on UC and exposit the molecular mechanism, we used the chemically induced experimental ulcer colitis model. DSS polymers were fed mice in the drinking water at the concentration of 3% (w/v) for 7 days. Bloody diarrhea, ulcerations, weight loss, and infiltrations with granulocytes which are mimic some clinical symptoms of inflammatory bowel diseases (Ge et al., 2016) appeared. Our data indicated that the experimental UC was successful with little mortality rates, and evodiamine attenuated colitis injury. Shen also found that evodiamine is able to ameliorate UC (Shen et al., 2019).

In patients with inflammatory bowel disease, a large population of inflammatory cells infiltrated in the mucosa, excessive inflammatory cytokines including tumor necrosis factor (TNF)-α, interleukin (IL)-18, IL-6, IL-1β were secreted (Wang et al., 2016; Bank et al., 2019). Especially, IL-1β and IL-18 are essential in the early phase of the inflammatory cascade (Liu et al., 2016); high levels of IL-1β revealed the severity of inflammation. To confirm the effect of evodiamine on UC, we detected the two critical inflammatory cytokines IL-1β and IL-18 in mice colon and THP1 cells stimulated by evodiamine. The two cytokines are both decreased by evodiamine in tissue and cells. IL-1β can be produced in NLRP3 inflammasome-dependent way or inflammasome-independent way (Davis et al., 2011; Netea et al., 2015). Shen et al. also showed that activity of NLRP3 inflammasome was inhibited by evodiamine in mice colonic tissues (Shen et al., 2019). Whereas Li et al. reported that evodiamine promoted NLRP3 inflammasome activation in J774A.1 and bone marrow-derived macrophages (BMDMs) (Li et al., 2019). Our data demonstrated that there was no significant alteration in NLRP3 expression. NLRP3 inflammasome was assembled by NLRP3, ASC and pro-caspase 1, we continued to examine the effect of evodiamine on inflammasome assembly.

NLRP3 are unable to assemble inflammasome complexes in the absence of ASC which is an essential adapter molecule linking NLRP3 to IL-1β for most inflammasome sensors (Monie, 2017). For NLRP3 inflammasome assembly, ASC might be ubiquitinated, it also forms another caspase-1-activating platform, called the ASC speck or pyroptosome (Fernandes-Alnemri et al., 2007; Guo et al., 2015). The ASC speck is an oligomer of ASC dimers. We determine ASC speck to evaluate the effect of evodiamine on the assembly of NLRP3. Our data demonstrated that ASC protein expression was diminished in mice colonic tissues and THP1 macrophage by evodiamine. Furthermore, the critical step of NLRP3 inflammasome activation (Chen L. et al., 2017), ASC oligomerization was decreased by evodiamine. Decreased colocalization of ASC and caspase1 indicated that NLRP3 inflammasome assembly was inhibited by evodiamine. We firstly reported that ASC oligomerization was able to be inhibited by evodiamine.

Owing to the critical role of NFκB in the progression of UC, we detected the effect of evodiamine on the activation of NFκB. In line with Shen’s work, we also found that evodiamine prevented the activity of key protein include p65 and IκB in NFκB pathway in UC mice.

Xu’s lab reported that NLRP3 inflammasome is mediated by mitophagy in colitis-associated cancer. To intervene autophagy and inflammasome at early stage is possible to prevent the cancer process (Guo et al., 2014). Currently, there is no clinical medicine effect on autophagy and NLRP3 inflammasome for the treatment of UC. Since evodiamine ameliorated colitis injury and inhibited NLRP3 inflammasome, inflammasome and autophagy interacts, we want to know whether evodiamine also act on autophagy. Then we examined p62, an LC3-binding adaptor protein, binds ubiquitinated substrates and transfers substrates to the autophagosome serving as a molecular bridge (Seibenhener et al., 2004). Accumulation of p62 in the cytoplasm indicated that autophagy was impaired and autophagic activity was reduced (Klionsky et al., 2016). We observed that p62 was upregulated in UC mice induced by DSS. It is consistent with Mimouna’s work that defective autophagic flux with elevated p62 in IBD patients (Mimouna et al., 2014).The decrement of P62 in evodiamine group suggested that autophagy is activated. Autophagy are related the conversion of LC3-I to LC3-II, especially, the amount of membrane-bound LC3-II provides a good index of autophagy induction (Kaminskyy et al., 2011; Klionsky et al., 2016). We continued to observe the effect of evodiamine on LC3-I and LC3-II. LC3-I protein conjugates to phosphatidylethanolamine and converted to LC3-II, which is translocated to the membrane of autophago- and autolysosomes. Our data demonstrated LC3-I and LC3-II were enhanced with the stimulation of DSS. In the early stage of ulcer colitis, the activation of autophagy attenuates the damage. We observed the levels of LC3-I and LC3-II were enhanced by evodiamine in Figure 5B. Thus, it indicated that autophagy intervened in the treatment of evodiamine for colitis.

There was no research on the effect of evodiamine on the link of NLRP3 inflammasome and autophagy. NLRP3 inflammasome is composed of NLRP3, ASC and caspase1. Shi et al. showed that ASC was polyubiquitinated with K63-linked chains and interacted with p62 upon inflammasome activation, suggesting that ASC can be targeted to autophagosomes (Shi et al., 2012). ASC is entirely enclosed by LC3 right structures in autophagy. James lab’s work also indicated that active inflammasomes can targeted to the autophagosome. Our data showed that evodiamine increased the colocalization of ASC and LC3 (Figure 5D). It suggested that evodiamine improved autophagy. Our confocal imaging further demonstrated that direct co-localization of ASC with LAMP1. Eventually, inflammasomes are delivered to lysosomes to destruct (Harris et al., 2017).

Our data indicate that evodiamine activates autophagy, leads to autophagosomal degradation of pro-inflammatory cytokine upstream of the NFκB signaling cascade. We found that evodiamine dramatically inhibited phosphorylated IκB and phosphorylated p65 in the colonic tissues of DSS-induced colitis mice. These results showed that evodiamine attenuated colitis via the induction of autophagosome-mediated degradation of inflammasome and the inhibition of NFκB pathway, which synergistically contribute to the effect of evodiamine in colitis. Yu et al. reviewed that evodiamine can act on multi-target including NO, COX-2 and so on in the inflammatory process (Yu et al., 2013). Herein, we did not determine other molecular targets and we cannot answer whether evodiamine can bind multi-target proteins involved in the inflammatory process or autophagy.

In summary, we showed that evodiamine ameliorated UC injury via the inhibition of NLRP3 inflammasome (Graphic Abstract). We firstly revealed that the suppression of NLRP3 activity induced by evodiamine was due to synergistically the induction of autophagosome-mediated degradation of inflammasome and the inhibition of NFκB pathway. Our data further demonstrated that evodiamine might act as a novel and potential drug for the therapy of acute colitis based on the effecting on autophagy and NLRP3 inflammasome. We expected that our study will offer directions toward colitis therapeutics.

## Data Availability Statement

The raw data supporting the conclusions of this article will be made available by the authors, without undue reservation.

## Ethics Statement

The animal study was reviewed and approved by Nanjing University Animal Welfare and Ethics committee.

## Author Contributions

RD conceived and designed the study. WD, ZD, YW, YZ, and QG performed the experiments. WD and RD wrote the paper. RD and WC reviewed and edited the manuscript. All authors read and approved the manuscript.

## Funding

This work was supported by the National Natural Science Foundation of China (Nos. 81673430, 81771539) and National Undergraduate Training Programs for Innovation (No. 201910284025).

## Conflict of Interest

The authors declare that the research was conducted in the absence of any commercial or financial relationships that could be construed as a potential conflict of interest.

## References

[B1] Bank S. Julsgaard M. Abed O. K. Burisch J. Broder Brodersen J. Pedersen N. K. (2019). Polymorphisms in the NFkB, TNF-alpha, IL-1beta, and IL-18 pathways are associated with response to anti-TNF therapy in Danish patients with inflammatory bowel disease. Aliment. Pharmacol. Ther. 49 (7), 890–903. 10.1111/apt.15187 30811631

[B2] Baumgart D. C. Sandborn W. J. (2007). Inflammatory bowel disease: clinical aspects and established and evolving therapies. Lancet 369 (9573), 1641–1657. 10.1016/s0140-6736(07)60751-x 17499606

[B3] Bryan N. B. Dorfleutner A. Rojanasakul Y. Stehlik C. (2009). Activation of inflammasomes requires intracellular redistribution of the apoptotic speck-like protein containing a caspase recruitment domain. J. Immunol. 182 (5), 3173–3182. 10.4049/jimmunol.0802367 19234215 PMC2652671

[B4] Chen H. Yang D. Han F. Tan J. Zhang L. Xiao J. (2017). The bacterial T6SS effector EvpP prevents NLRP3 inflammasome activation by inhibiting the Ca(2+)-dependent MAPK-jnk pathway. Cell Host Microbe 21 (1), 47–58. 10.1016/j.chom.2016.12.004 28081443

[B5] Chen L. You Q. Hu L. Gao J. Meng Q. Liu W. (2017). The antioxidant procyanidin reduces reactive oxygen species signaling in macrophages and ameliorates experimental colitis in mice. Front. Immunol. 8, 1910 10.3389/fimmu.2017.01910 29354126 PMC5760499

[B6] Davis B. K. Wen H. Ting J. P. (2011). The inflammasome NLRs in immunity, inflammation, and associated diseases. Annu. Rev. Immunol. 29, 707–735. 10.1146/annurev-immunol-031210-101405 21219188 PMC4067317

[B7] Deretic V. Saitoh T. Akira S. (2013). Autophagy in infection, inflammation and immunity. Nat. Rev. Immunol. 13 (10), 722–737. 10.1038/nri3532 24064518 PMC5340150

[B8] Dupont N. Jiang S. Pilli M. Ornatowski W. Bhattacharya D. Deretic V. (2011). Autophagy-based unconventional secretory pathway for extracellular delivery of IL-1beta. EMBO J. 30 (23), 4701–4711. 10.1038/emboj.2011.398 22068051 PMC3243609

[B9] Fernandes-Alnemri T. Wu J. Yu J. W. Datta P. Miller B. Jankowski W. (2007). The pyroptosome: a supramolecular assembly of ASC dimers mediating inflammatory cell death via caspase-1 activation. Cell Death Differ. 14 (9), 1590–1604. 10.1038/sj.cdd.4402194 17599095 PMC3345951

[B10] Galli S. J. Borregaard N. Wynn T. A. (2011). Phenotypic and functional plasticity of cells of innate immunity: macrophages, mast cells and neutrophils. Nat. Immunol. 12 (11), 1035–1044. 10.1038/ni.2109 22012443 PMC3412172

[B11] Ganley I. G. Wong P. M. Gammoh N. Jiang X. (2011). Distinct autophagosomal-lysosomal fusion mechanism revealed by thapsigargin-induced autophagy arrest. Mol. Cell 42 (6), 731–743. 10.1016/j.molcel.2011.04.024 21700220 PMC3124681

[B12] Ge X. Chen S. Y. Liu M. Liang T. M. Liu C. (2016). Evodiamine inhibits PDGFBBinduced proliferation of rat vascular smooth muscle cells through the suppression of cell cycle progression and oxidative stress. Mol. Med. Rep. 14 (5), 4551–4558. 10.3892/mmr.2016.5798 27748810 PMC5101993

[B13] Guo W. Sun Y. Liu W. Wu X. Guo L. Cai P. (2014). Small molecule-driven mitophagy-mediated NLRP3 inflammasome inhibition is responsible for the prevention of colitis-associated cancer. Autophagy 10 (6), 972–985. 10.4161/auto.28374 24879148 PMC4091180

[B14] Guo H. Callaway J. B. Ting J. P. (2015). Inflammasomes: mechanism of action, role in disease, and therapeutics. Nat. Med. 21 (7), 677–687. 10.1038/nm.3893 26121197 PMC4519035

[B15] Hamasaki M. Yoshimori T. (2010). Where do they come from? Insights into autophagosome formation. FEBS Lett. 584 (7), 1296–1301. 10.1016/j.febslet.2010.02.061 20188731

[B16] Harris J. Lang T. Thomas J. P. W. Sukkar M. B. Nabar N. R. Kehrl J. H. (2017). Autophagy and inflammasomes. Mol. Immunol. 86, 10–15. 10.1016/j.molimm.2017.02.013 28249679

[B17] Hayashi S. Hamada T. Zaidi S. F. Oshiro M. Lee J. Yamamoto T. (2014). Nicotine suppresses acute colitis and colonic tumorigenesis associated with chronic colitis in mice. Am. J. Physiol. Gastrointest. Liver Physiol. 307 (10), G968–G978. 10.1152/ajpgi.00346.2013 25258409

[B18] Jin C. Flavell R. A. (2010). Molecular mechanism of NLRP3 inflammasome activation. J. Clin. Immunol. 30 (5), 628–631. 10.1007/s10875-010-9440-3 20589420

[B19] Kaminskyy V. Abdi A. Zhivotovsky B. (2011). A quantitative assay for the monitoring of autophagosome accumulation in different phases of the cell cycle. Autophagy 7 (1), 83–90. 10.4161/auto.7.1.13893 20980814

[B20] Kim M. J. Yoon J. H. Ryu J. H. (2016). Mitophagy: a balance regulator of NLRP3 inflammasome activation. BMB Rep. 49 (10), 529–535. 10.5483/bmbrep.2016.49.10.115 27439607 PMC5227293

[B21] Klionsky D. J. Abdelmohsen K. Abe A. Abedin M. J. Abeliovich H. Acevedo Arozena A. (2016). Guidelines for the use and interpretation of assays for monitoring autophagy (3rd edition). Autophagy 12 (1), 1–222. 10.1080/15548627.2015.1100356 26799652 PMC4835977

[B22] Li C. G. Zeng Q. Z. Chen M. Y. Xu L. H. Zhang C. C. Mai F. Y. (2019). Evodiamine augments NLRP3 inflammasome activation and anti-bacterial responses through inducing alpha-tubulin acetylation. Front. Pharmacol. 10, 290 10.3389/fphar.2019.00290 30971927 PMC6443907

[B23] Liu A. J. Wang S. H. Chen K. C. Kuei H. P. Shih Y. L. Hou S. Y. (2013). Evodiamine, a plant alkaloid, induces calcium/JNK-mediated autophagy and calcium/mitochondria-mediated apoptosis in human glioblastoma cells. Chem. Biol. Interact. 205 (1), 20–28. 10.1016/j.cbi.2013.06.004 23774672

[B24] Liu W. Guo W. Wu J. Luo Q. Tao F. Gu Y. (2013). A novel benzo[d]imidazole derivate prevents the development of dextran sulfate sodium-induced murine experimental colitis via inhibition of NLRP3 inflammasome. Biochem. Pharmacol. 85 (10), 1504–1512. 10.1016/j.bcp.2013.03.008 23506741

[B25] Liu X. Zhou W. Zhang X. Lu P. Du Q. Tao L. (2016). Dimethyl fumarate ameliorates dextran sulfate sodium-induced murine experimental colitis by activating Nrf2 and suppressing NLRP3 inflammasome activation. Biochem. Pharmacol. 112, 37–49. 10.1016/j.bcp.2016.05.002 27184504

[B26] Lorden G. Sanjuan-Garcia I. de Pablo N. Meana C. Alvarez-Miguel I. Perez-Garcia M. T. (2017). Lipin-2 regulates NLRP3 inflammasome by affecting P2X7 receptor activation. J. Exp. Med. 214 (2), 511–528. 10.1084/jem.20161452 28031477 PMC5294860

[B27] Lu A. Magupalli V. G. Ruan J. Yin Q. Atianand M. K. Vos M. R. (2014). Unified polymerization mechanism for the assembly of ASC-dependent inflammasomes. Cell 156 (6), 1193–1206. 10.1016/j.cell.2014.02.008 24630722 PMC4000066

[B28] Ma Y. Galluzzi L. Zitvogel L. Kroemer G. (2013). Autophagy and cellular immune responses. Immunity 39 (2), 211–227. 10.1016/j.immuni.2013.07.017 23973220

[B29] Mei Y. Fang C. Ding S. Liu X. Hu J. Xu J. (2019). PAP-1 ameliorates DSS-induced colitis with involvement of NLRP3 inflammasome pathway. Int. Immunopharm. 75, 105776 10.1016/j.intimp.2019.105776 31351364

[B30] Mimouna S. Bazin M. Mograbi B. Darfeuille-Michaud A. Brest P. Hofman P. (2014). HIF1A regulates xenophagic degradation of adherent and invasive *Escherichia coli* (AIEC). Autophagy 10 (12), 2333–2345. 10.4161/15548627.2014.984275 25484075 PMC4502747

[B31] Monie T. P. (2017). The canonical inflammasome: a macromolecular complex driving inflammation. Subcell. Biochem. 83, 43–73. 10.1007/978-3-319-46503-6_2 28271472

[B32] Nakahira K. Haspel J. A. Rathinam V. A. Lee S. J. Dolinay T. Lam H. C. (2011). Autophagy proteins regulate innate immune responses by inhibiting the release of mitochondrial DNA mediated by the NALP3 inflammasome. Nat. Immunol. 12 (3), 222–230. 10.1038/ni.1980 21151103 PMC3079381

[B33] Netea M. G. van de Veerdonk F. L. van der Meer J. W. Dinarello C. A. Joosten L. A. (2015). Inflammasome-independent regulation of IL-1-family cytokines. Annu. Rev. Immunol. 33, 49–77. 10.1146/annurev-immunol-032414-112306 25493334

[B34] Okada M. Matsuzawa A. Yoshimura A. Ichijo H. (2014). The lysosome rupture-activated TAK1-JNK pathway regulates NLRP3 inflammasome activation. J. Biol. Chem. 289 (47), 32926–32936. 10.1074/jbc.M114.579961 25288801 PMC4239639

[B35] Okumura R. Kurakawa T. Nakano T. Kayama H. Kinoshita M. Motooka D. (2016). Lypd8 promotes the segregation of flagellated microbiota and colonic epithelia. Nature 532 (7597), 117–121. 10.1038/nature17406 27027293

[B36] Qin T. Du R. Huang F. Yin S. Yang J. Qin S. (2016). Sinomenine activation of Nrf2 signaling prevents hyperactive inflammation and kidney injury in a mouse model of obstructive nephropathy. Free Radic. Biol. Med. 92, 90–99. 10.1016/j.freeradbiomed.2016.01.011 26795599

[B37] Rogler G. (2012). Inflammatory bowel disease cancer risk, detection and surveillance. Dig. Dis. 30 (Suppl. 2), 48–54. 10.1159/000341893 23207932

[B38] Ryter S. W. Cloonan S. M. Choi A. M. (2013). Autophagy: a critical regulator of cellular metabolism and homeostasis. Mol. Cell 36 (1), 7–16. 10.1007/s10059-013-0140-8 PMC3887921 23708729

[B39] Salim S. Y. Soderholm J. D. (2011). Importance of disrupted intestinal barrier in inflammatory bowel diseases. Inflamm. Bowel Dis. 17 (1), 362–381. 10.1002/ibd.21403 20725949

[B40] Seibenhener M. L. Babu J. R. Geetha T. Wong H. C. Krishna N. R. Wooten M. W. (2004). Sequestosome 1/p62 is a polyubiquitin chain binding protein involved in ubiquitin proteasome degradation. Mol. Cell Biol. 24 (18), 8055–8068. 10.1128/MCB.24.18.8055-8068.2004 15340068 PMC515032

[B41] Shen P. Zhang Z. Zhu K. Cao H. Liu J. Lu X. (2019). Evodiamine prevents dextran sulfate sodium-induced murine experimental colitis via the regulation of NF-kappaB and NLRP3 inflammasome. Biomed. Pharmacother. 110, 786–795. 10.1016/j.biopha.2018.12.033 30554117

[B42] Shi C. S. Shenderov K. Huang N. N. Kabat J. Abu-Asab M. Fitzgerald K. A. (2012). Activation of autophagy by inflammatory signals limits IL-1beta production by targeting ubiquitinated inflammasomes for destruction. Nat. Immunol. 13 (3), 255–263. 10.1038/ni.2215 22286270 PMC4116819

[B43] Song M. Park H. J. (2014). Anti-inflammatory effect of *Phellinus linteus* grown on germinated brown rice on dextran sodium sulfate-induced acute colitis in mice and LPS-activated macrophages. J. Ethnopharmacol. 154 (2), 311–318. 10.1016/j.jep.2013.12.059 24495471

[B44] Sun Y. Zhao Y. Yao J. Zhao L. Wu Z. Wang Y. (2015). Wogonoside protects against dextran sulfate sodium-induced experimental colitis in mice by inhibiting NF-kappaB and NLRP3 inflammasome activation. Biochem. Pharmacol. 94 (2), 142–154. 10.1016/j.bcp.2015.02.002 25677765

[B45] Tasaka Y. Yasunaga D. Kiyoi T. Tanaka M. Tanaka A. Suemaru K. (2015). Involvement of stimulation of alpha7 nicotinic acetylcholine receptors in the suppressive effect of tropisetron on dextran sulfate sodium-induced colitis in mice. J. Pharmacol. Sci. 127 (3), 275–283. 10.1016/j.jphs.2014.12.016 25837923

[B46] Thomas N. B. Hutcheson I. R. Campbell L. Gee J. Taylor K. M. Nicholson R. I. (2010). Growth of hormone-dependent MCF-7 breast cancer cells is promoted by constitutive caveolin-1 whose expression is lost in an EGF-R-mediated manner during development of tamoxifen resistance. Breast Cancer Res. Treat. 119 (3), 575–591. 10.1007/s10549-009-0355-8 19288272

[B47] Ting J. P. Lovering R. C. Alnemri E. S. Bertin J. Boss J. M. Davis B. K. (2008). The NLR gene family: a standard nomenclature. Immunity 28 (3), 285–287. 10.1016/j.immuni.2008.02.005 18341998 PMC2630772

[B48] Tursi A. Brandimarte G. Papa A. Giglio A. Elisei W. Giorgetti G. M. (2010). Treatment of relapsing mild-to-moderate ulcerative colitis with the probiotic VSL#3 as adjunctive to a standard pharmaceutical treatment: a double-blind, randomized, placebo-controlled study. Am. J. Gastroenterol. 105 (10), 2218–2227. 10.1038/ajg.2010.218 20517305 PMC3180711

[B49] Wang S. Fang K. Dong G. Chen S. Liu N. Miao Z. (2015). Scaffold diversity inspired by the natural product evodiamine: discovery of highly potent and multitargeting antitumor agents. J. Med. Chem. 58 (16), 6678–6696. 10.1021/acs.jmedchem.5b00910 26226379

[B50] Wang Y. Wang H. Qian C. Tang J. Zhou W. Liu X. (2016). 3-(2-Oxo-2-phenylethylidene)-2,3,6,7-tetrahydro-1H-pyrazino[2,1-a]isoquinolin-4(11bH)-one (compound 1), a novel potent Nrf2/ARE inducer, protects against DSS-induced colitis via inhibiting NLRP3 inflammasome. Biochem. Pharmacol. 101, 71–86. 10.1016/j.bcp.2015.11.015 26588861

[B51] Wei W. Ding M. Zhou K. Xie H. Zhang M. Zhang C. (2017). Protective effects of wedelolactone on dextran sodium sulfate induced murine colitis partly through inhibiting the NLRP3 inflammasome activation via AMPK signaling. Biomed. Pharmacother. 94, 27–36. 10.1016/j.biopha.2017.06.071 28750357

[B52] Wu X. Guo W. Wu L. Gu Y. Gu L. Xu S. (2012). Selective sequestration of STAT1 in the cytoplasm via phosphorylated SHP-2 ameliorates murine experimental colitis. J. Immunol. 189 (7), 3497–3507. 10.4049/jimmunol.1201006 22942432

[B53] Xia Y. Liu N. Xie X. Bi G. Ba H. Li L. (2019). The macrophage-specific V-ATPase subunit ATP6V0D2 restricts inflammasome activation and bacterial infection by facilitating autophagosome-lysosome fusion. Autophagy 15 (6), 960–975. 10.1080/15548627.2019.1569916 30681394 PMC6526827

[B54] Yu H. Jin H. Gong W. Wang Z. Liang H. (2013). Pharmacological actions of multi-target-directed evodiamine. Molecules 18 (2), 1826–1843. 10.3390/molecules18021826 23434865 PMC6270287

[B55] Yuk J. M. Jo E. K. (2013). Crosstalk between autophagy and inflammasomes. Mol. Cell 36 (5), 393–399. 10.1007/s10059-013-0298-0 PMC3887939 24213677

[B56] Zaki M. H. Boyd K. L. Vogel P. Kastan M. B. Lamkanfi M. Kanneganti T. D. (2010). The NLRP3 inflammasome protects against loss of epithelial integrity and mortality during experimental colitis. Immunity 32 (3), 379–391. 10.1016/j.immuni.2010.03.003 20303296 PMC2982187

[B57] Zaki M. H. Lamkanfi M. Kanneganti T. D. (2011). The Nlrp3 inflammasome: contributions to intestinal homeostasis. Trends Immunol. 32 (4), 171–179. 10.1016/j.it.2011.02.002 21388882 PMC3070791

[B58] Zhao Z. Gong S. Wang S. Ma C. (2015). Effect and mechanism of evodiamine against ethanol-induced gastric ulcer in mice by suppressing Rho/NF-small ka, CyrillicB pathway. Int. Immunopharm. 28 (1), 588–595. 10.1016/j.intimp.2015.07.030 26225926

